# Nontuberculous mycobacterial infection and environmental molybdenum in persons with cystic fibrosis: a case–control study in Colorado

**DOI:** 10.1038/s41370-021-00360-2

**Published:** 2021-07-03

**Authors:** Ettie M. Lipner, James L. Crooks, Joshua French, Michael Strong, Jerry A. Nick, D. Rebecca Prevots

**Affiliations:** 1 Center for Genes, Environment and Health, National Jewish Health, Denver, CO USA; 2 Department of Epidemiology, Colorado School of Public Health, Aurora, CO USA; 3 Division of Biostatistics and Bioinformatics, National Jewish Health, Denver, CO USA; 4 Department of Mathematical and Statistical Sciences, University of Colorado Denver, Denver, CO USA; 5 Department of Medicine, National Jewish Health, Denver, CO USA; 6 National Institute of Allergy and Infectious Diseases, National Institutes of Health, Bethesda, MD USA

**Keywords:** Nontuberculous mycobacteria, Molybdenum, Case–control study, Cystic fibrosis, Water quality, Geospatial

## Abstract

**Rationale:**

Nontuberculous mycobacteria (NTM) are ubiquitous environmental bacteria that may cause chronic lung disease and are one of the most difficult-to-treat infections among persons with cystic fibrosis (pwCF). Environmental factors likely contribute to increased NTM densities, with higher potential for exposure and infection.

**Objective:**

To identify water-quality constituents that influence odds of NTM infection among pwCF in Colorado.

**Methods:**

We conducted a population-based nested case–control study using patient data from the Colorado CF Center NTM database. We associated data from pwCF and water-quality data extracted from the Water Quality Portal to estimate odds of NTM infection. Using Bayesian generalized linear models with binomial-distributed discrete responses, we modeled three separate outcomes; any NTM infection, infections due to *Mycobacterium avium* complex species, and infections due to *M. abscessus* group species.

**Results:**

We observed a consistent association with molybdenum in the source water and *M. abscessus* group species infection among pwCF in all models. For every 1-unit increase in the log concentration of molybdenum in surface water, the odds of infection for those with *M. abscessus* group species compared to those who were NTM culture-negative increased by 79%. The odds of *M. abscessus* group infection varied by county; the counties with the highest probability of infection are located along the major rivers.

**Conclusions:**

We have identified molybdenum in the source water as the most predictive factor of *M. abscessus* group infection among pwCF in Colorado. This finding will help inform patients at risk for NTM of their relative risks in residing within specific regions.

## Introduction

Pulmonary nontuberculous mycobacterial (NTM) disease among persons with cystic fibrosis (pwCF) is challenging to treat, requiring prolonged treatment courses [1]. Over a recent 5-year interval, nearly 20% of children and adults with CF in the United States who were tested had positive cultures for NTM, of whom 39% had infections with *Mycobacterium abscessus* [2], which is one of the most difficult-to-treat NTM species [3]. Distinct geographic variability of NTM disease has been demonstrated in both general and CF populations [2, 4, 5]. Environmental determinants of NTM infection and disease include factors related to moisture in the environment, as well as soil [6] and soil components [4, 7, 8]. However, the sources of NTM infection and exposure risks are poorly understood. Environmental conditions related to soil properties, natural water, and engineered water system characteristics, including biofilm formation in premise plumbing, likely contribute to increased NTM densities with higher potential for NTM exposure and infection. Prevention of infections with NTM among pwCF is a critical clinical need [9].

In two previous studies, we explored the role of water exposure in NTM risk. We identified three high-risk watersheds in Colorado [10], and further used source water data [11] to identify factors potentially influencing the higher risk in these watershed regions. Molybdenum in surface water was a significant contributor to the risk of NTM infection; a 1-unit increase in the log concentration of molybdenum in surface water was associated with a 17% increased risk of NTM infection. Research to date suggests a physiological connection linking molybdenum and essential metabolism of *M. tuberculosis*, a phylogenetically related organism to NTM, potentially impacting survival, pathogenesis, and persistence [12–14]. Given the genetic relatedness of *M. tuberculosis* and NTM, we hypothesize that higher concentrations of specific water-quality constituents, potentially molybdenum, which the bacteria may require for metabolism and growth, result in higher densities of NTM in surface water sources in certain regions. Thus, infection rates would be higher in regions with a water supply from sources with high densities of NTM. In our current study, we hypothesize that specific water-quality constituents in surface water in Colorado influence the odds of having NTM infection among pwCF. To test this hypothesis, we conducted a nested case–control study using water-quality data from the Water Quality Portal (WQP), sponsored by the U.S. Geological Survey, U.S. Environmental Protection Agency, and National Water Quality Monitoring Council, together with CF patient data extracted from the Colorado CF Center NTM database.

## Methods

### Data collection

#### Study design and subjects

This study was a nested case–control study using demographic and clinical data from the Colorado CF Center NTM database. The Colorado CF Center comprises the Pediatric CF Program at The Children’s Hospital Colorado in Aurora, Colorado, and an Adult CF Program at National Jewish Health in Denver, Colorado. The Colorado CF Center is the only CF Center in the state and has nearly complete capture of all CF patients in Colorado. This study therefore can be described as a population-based CF study.

The Colorado CF Center NTM database contained data on pwCF resident in Colorado from January 2007 through January 2019. We extracted patient ZIP code, NTM species, and demographic information. Because we did not have patient address information and our data were too sparse at the ZIP code level, we aggregated all patient ZIP codes to the county level. Cases were defined as CF patients who had at least one positive NTM culture and were resident in Colorado at the time of their first positive culture, as determined by chart review. We excluded CF patients who had cultured positive only for *M. gordonae* infection. Controls were defined as patients with CF who had at least three negative cultures within a single county over a period of at least 3 years (“NTM-negative”). Our study population comprised 388 CF patients; 193 cases and 195 controls. This study was approved by the NJH Institutional Review Board (HS-1683).

#### NTM species

Frequencies of NTM species from patient isolates are listed in Supplementary Table 1. Molecular assays by Line Probe Assay analysis or targeted gene sequencing were used to differentiate *Mycobacterium* species. NTM identification was performed by the Advanced Diagnostics laboratory at NJH, a National Reference Laboratory for NTM.

#### Water-quality data compilation

We obtained water-quality data from the WQP [15], a water-quality database collected or hosted by the U.S. Geological Survey, the U.S. Environmental Protection Agency, and the National Water Quality Monitoring Council. Our water-quality dataset has been described previously [11]. Supplementary Table 2 presents the median and standard deviation values of the water-quality constituents obtained from the WQP that were used in our analyses.

### Statistical analysis

**Fig. 1:**
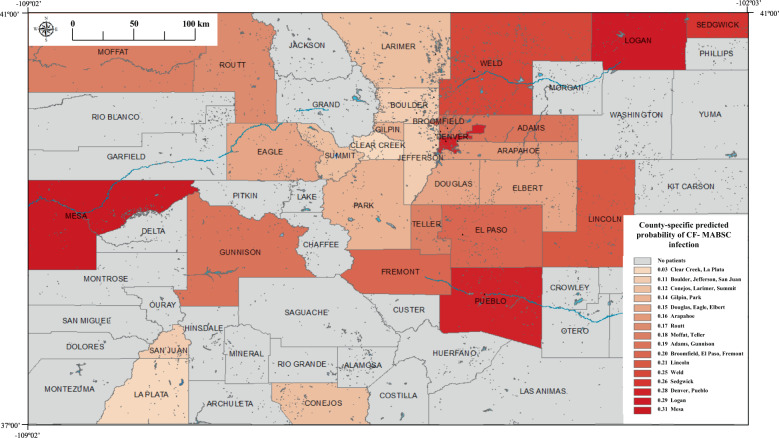
Predicted probability of *M*. *abscessus* infection for counties where pwCF resided. Gray lines represent county line boundaries in Colorado. County names are printed in black. Blue areas indicate lakes, reservoirs, and rivers.

All water-sample sites were aggregated by county. Subsequently, we calculated the median value of each water-quality constituent for each county. Apparent concentration-unit reporting errors were corrected (for example, three orders of magnitude deviations for individual values were multiplied by 1000 to align them with the range of the remaining source-specific data). Water-quality constituents were eliminated if data were not available for more than 50% of counties. Following these curation steps, 17 remaining water-quality constituents remained for analysis (Supplementary Table 2). We used a natural log transformation of all county-median variables (17 variables). We standardized all the water-quality constituents’ log concentrations to have a mean of 0 and standard deviation of 1. For counties with missing data, we imputed the median value of all water-quality constituents. We also calculated drive-time between county centroids and NJH. For patients with any NTM infection, 31 counties were dropped from the analysis because there was not at least one case or one control resident in those counties, with 33 remaining counties (51.6%) available for analysis. For patients with *M. avium* complex (MAC) and *M. abscessus* infection, 31 counties (48.4%) and 29 counties (45.3%), respectively, were available for analysis. Each patient was assigned the water-quality value for his or her respective county of residence. The counties with available data are shown with non-gray coloring in Fig. 1.

#### Variable reduction using principal component analysis (PCA)

Principal component analysis (PCA) was used to reduce the number of predictors considered in our subsequent models. PCA is used to determine orthogonal “components” that explain the most variation in the data, where each component is a weighted combination of the predictor variables. For the components explaining the most variation, the variables with the most weight in these components were identified for use in future models. PCA was performed on 17 water-quality constituents summarized at the county level (after these values were natural log transformed, scaled, and imputed).

Principal components 1 and 2 explained 58.9% of the data variability. Any constituents in the first two components that had a greater contribution than what is expected under equal contribution were identified as important contributors [16, 17]. This process is illustrated graphically in Supplementary Fig. 1, where the dashed red line represents what is expected under equal contribution. This threshold captured 11 out of 17 constituents: cadmium, calcium, chloride, magnesium, molybdenum, manganese, potassium, selenium, sodium, sulfate, and zinc.

#### Parameters used in Bayesian binomial regression models

We used Bayesian generalized linear models (GLMs) to model the relationship between NTM infection and demographic and water quality variables. In these models, the dependent variable is NTM infection status, and the predictors are demographic and water quality variables. Diagnostic tools were used to confirm that the fitted models adequately represented the observed pattern of the data. Because age, sex, and race\ethnicity are associated with the risk of NTM infection [2, 18, 19], and could also influence county of residence, we included these as confounders in our model. These relationships are depicted in a Directed Acyclic Graph in Supplementary Fig. 2.

For each subject, county-level median values of each water-quality constituent (standardized, imputed) were included. In addition, we included a binary variable indicating whether a county’s centroid center was within a 1-h drive to NJH. To control for a higher proportion of patients residing in counties located in the Front Range with greater access to treatment, we categorized counties based on whether their centroid center was within a 1.0-h drive to NJH. We also performed sensitivity analyses to exclude the drive-time variable from our models (Supplementary Table 3).

#### Bayesian binomial regression models with individual metals from principal components 1 and 2

We modeled three separate outcomes (any NTM infection, infections due to MAC species, and infections due to *M. abscessus* group species as a function of water-quality constituents and demographic variables (Supplementary Table 4). Then, for each outcome, we constructed a subsequent model (Model 1) that included only those water-quality constituents whose variance inflation factor was less than 10 to mitigate the potential impact of collinear covariates. For the three models, we sequentially removed the constituent with the highest variance inflation factor. The constituents with variance inflation factors over 10 included magnesium, sodium, potassium, and sulfate, resulting in a final model (Model 1) with the following water-quality constituents: cadmium, calcium, chloride, manganese, molybdenum, selenium, zinc. The correlation matrix for water-quality constituents are shown in Supplementary Table 5. Finally, we constructed separate single-constituent Bayesian GLMs for the water-quality constituents that were significant in Model 1 (as assessed by having a 90% central credible interval (CI) that did not include 1) (Model 2). We estimated the odds of NTM infection among pwCF given exposure to water-quality constituents in surface water sources.

We present an odds ratio and 90% central CI for each model variable. CIs were used to assess the posterior probability of an association between each model variable and a change in the odds of NTM infection and 90% CIs were reported owing to greater computational stability than the 95% CIs in the rstanarm package [20].

We predicted the probability that an unobserved CF patient living in a county will have an NTM infection and displayed the results as a probability map across Colorado counties (Fig. 1). The software used to perform the analysis are discussed in the Supplementary Materials. Reproducible source code for the analyses is also provided in the Supplementary Materials.

## Results

### Study population characteristics

Our study population comprised pwCF who received medical care at the Colorado CF Center, and included 195 CF NTM culture-negative patients and 193 pwCF who had at least one positive culture, of whom 147 (76.2%) had MAC infection (*M. avium, M. intracellulare, M. chimaera*) and 82 (42.3%) had *M. abscessus* complex infection (*M. abscessus/chelonae, M. massiliense, M. bolletti*). Forty-six (23.7%) patients had both MAC and *M. abscessus* infections at any time. Patients with both MAC and *M. abscessus* infections were included in both subsets of patients. Demographic characteristics of cases and controls are shown in Table 1. We observed a younger mean age and a higher proportion of males among pwCF with *M. abscessus* infection compared to those with MAC infection. Given well-understood growth rate differences [21], distinct ecological niches [22], and specialized medical treatments [23] for MAC and *M. abscessus* infections, we modeled three separate outcomes: any NTM infection, infections due to MAC species, and infections due to *M. abscessus* species.

**Table 1:** Descriptive statistics of cases (NTM culture positive) and controls among a Colorado CF patient population.

Characteristic	Controls (CF only) *n* = 195	Patient infection from all NTM species *n* = 193	Patient infection from MAC species *n* = 147	Patient infection from MABSC species *n* = 82
Age, years, mean ± SD	35.66 ± 11.90	37.30 ± 13.37	37.66 ± 13.93	35.20 ± 10.80
Female sex, *n* (%)	95 (48.7)	109 (55.9)	86 (58.5)	36 (43.9)
White race, *n* (%)	187 (95.9)	187 (96.9)	143 (97.3)	80 (97.6)

### Bayesian binomial regression models with individual metals from principal components 1 and 2

Molybdenum was the only constituent significantly associated with increased odds of infection (i.e., 90% CI failed to include 1) (Table 2; Model 1). The results of these models indicate that for every 1-log unit increase in molybdenum concentrations in surface water, the odds of having NTM infection is 1.7, 1.9, and 2.5 times higher for infections caused by all NTM species, MAC species, and *M. abscessus* species, respectively, after controlling for other water-quality constituents.

**Table 2:** Model 1. Bayesian binomial regression model examining water-quality constituents (with VIF values less than 10 from Model 1) associated with odds of NTM infection among pwCF.

All NTM species		MAC species		MABSC species	
Variable	Odds ratio (90% CI)	Variable	Odds ratio (90% CI)	Variable	Odds ratio (90% CI)
Age: 1 year	1.01 (1.00, 1.03)	Age: 1 year	1.01 (1.00, 1.03)	Age: 1 year	1.00 (0.98, 1.03)
Gender: male	0.77 (0.54, 1.11)	Gender: male	0.70 (0.48, 1.03)	Gender: male	1.33 (0.84, 2.10)
Race: non-white^a^	0.75 (0.29, 1.93)	Race: non-white^a^	0.72 (0.21, 1.93)	Race: non-white^a^	0.40 (0.07, 1.43)
Drive-time (>1.0 h to NJH)	1.52 (0.90, 2.56)	Drive-time (>1.0 h to NJH)	1.67 (0.96, 2.92)	Drive-time (>1.0 h to NJH)	1.93 (0.93, 4.10)
Cadmium (1-log unit)	1.15 (0.84, 1.57)	Cadmium (1-log unit)	1.20 (0.86, 1.68)	Cadmium (1-log unit)	1.22 (0.80, 1.88)
Calcium (1-log unit)	0.89 (0.54, 1.45)	Calcium (1-log unit)	0.80 (0.47, 1.36)	Calcium (1-log unit)	0.54 (0.25, 1.13)
Chloride (1-log unit)	1.05 (0.73, 1.51)	Chloride (1-log unit)	1.06(0.72, 1.60)	Chloride (1-log unit)	1.10 (0.67, 1.84)
Manganese (1-log unit)	0.88 (0.57, 1.30)	Manganese (1-log unit)	0.98 (0.64, 1.52)	Manganese (1-log unit)	0.84 (0.45, 1.49)
Molybdenum (1-log unit)	**1.69 (1.04, 2.80)**	Molybdenum (1-log unit)	**1.87 (1.09, 3.25)**	Molybdenum (1-log unit)	**2.47 (1.28, 4.90)**
Selenium (1-log unit)	0.85 (0.54, 1.32)	Selenium (1-log unit)	0.81 (0.51, 1.28)	Selenium (1-log unit)	1.16 (0.61, 2.25)
Zinc (1-log unit)	1.37 (0.82, 2.32)	Zinc (1-log unit)	1.00 (0.57, 1.75)	Zinc (1-log unit)	2.14 (0.99, 5.42)

*CI* credible interval.

Bolded estimates have 90% CIs that fail to include 1.

^a^Reference group is white alone.

We then examined the 90% CI for exponentiated parameters of Model 1. The parameters whose 90% CI failed to include 1 were included in separate single-constituent models (Table 3; Model 2). For All NTM species and *M. abscessus* species, the CIs for molybdenum were entirely above 1, indicating a significantly higher odds of infection. Even more convincingly, the posterior probability that the molybdenum coefficient is positive (i.e., associated with increased odds of NTM for pwCF) is 96.96% for All NTM species, 94.15% for MAC species, and 99.96% for *M. abscessus* species (Supplementary Table 6). Our results indicate that for every 1-log unit increase in molybdenum concentration in surface water sources at the county level, the odds of having NTM infection caused by *M. abscessus* species increased by 79% compared with pwCF who were NTM-negative. When modeling all NTM species, we observed a weaker association for molybdenum. We did not observe an association between molybdenum and MAC infections. We also estimated these associations without including drive-time in the models (Supplementary Table 3). The association that we observed between molybdenum and *M. abscessus* infections remained significant (OR = 1.60), although slightly attenuated compared to our main results (OR = 1.79). The association between molybdenum and all NTM infections did not retain significance without including drive-time. Therefore, our results indicate that increasing concentrations of molybdenum in surface water increases the odds of *M. abscessus* infection.

**Table 3:** Model 2. Single-exposure Bayesian binomial regression model examining significant metals from Model 1 associated with odds of NTM infection among pwCF.

All NTM species		MAC species		MABSC species	
Variable	Odds ratio (90% CI)	Variable	Odds ratio (90% CI)	Variable	Odds ratio (90% CI)
Age: 1 year	1.01 (1.00, 1.02)	Age: 1 year	1.01 (1.00, 1.02)	Age: 1 year	1.01 (0.98, 1.02)
Gender: male	0.76 (0.54, 1.06)	Gender: male	**0.68** **(0.47, 0.99)**	Gender: male	1.21 (0.77, 1.92)
Race: non-white^a^	0.76 (0.29, 1.95)	Race: non-White^a^	0.67 (0.21, 1.88)	Race: non-White^a^	0.76 (0.09, 1.49)
Drive-time (>1.0 h to NJH)	1.28 (0.88, 1.92)	Drive-time (>1.0 h to NJH)	1.32 (0.89, 1.99)	Drive-time (>1.0 h to NJH)	**1.28** **(1.05, 2.75)**
Molybdenum (1-log unit)	**1.29** **(1.03, 1.62)**	Molybdenum (1-log unit)	1.26 (0.99, 1.61)	Molybdenum (1-log unit)	**1.79** **(1.34, 2.44)**

*CI* credible interval.

Bolded estimates have 90% CIs that fail to include 1.

^a^Reference group is white alone.

In Fig. 1, we calculated the predicted probability that an unobserved pwCF living in a county will have a *M. abscessus* infection based on a model using molybdenum as an independent predictor while controlling for drive-time, age race, and gender. The counties with the highest probability of *M. abscessus* infection are located along the major rivers; the South Platte River flowing through Denver, Logan, Sedgwick, and Weld counties, the Colorado River flowing through Mesa county, and the Arkansas River flowing through Pueblo county.

## Discussion

We found that molybdenum in surface water sources was associated with increased odds of NTM infection among pwCF, specifically for those with *M. abscessus* group infections. For every 1-log unit increase in molybdenum concentration in surface water among pwCF, the odds of NTM infection caused by *M. abscessus* species increased by 79% compared with those who were NTM-negative (Table 3; Model 2).

As discussed previously [11], molybdenum is involved in the essential metabolism of *M. tuberculosis* [12–14], and, given the genetic relatedness of these organisms, it is biologically plausible that it may play a similar role in NTM metabolism [24]. In this study, we replicated the molybdenum-NTM infection association in a CF population with water-quality constituent median values calculated for county line boundaries (instead of watershed boundaries [11]). This study also goes a step further to suggest that molybdenum in surface water may increase the odds of acquiring NTM, specifically for *M. abscessus* infection, rather than for MAC infection, in a CF population.

Molybdenum may promote NTM growth in surface water, thereby increasing the risk of exposure and infection. Because we did not have access to environmentally measured NTM densities, we used infection prevalence as a proxy for NTM abundance, assuming that higher NTM abundance increases the risk of NTM exposure and infection. A recent study [25] demonstrated that NTM abundance from premise plumbing samples as measured by 16S rRNA gene sequencing approach was significantly correlated with higher disease prevalence in population-based epidemiological studies [4]. This approach assumes that regions with high disease prevalence correlate with regions of high NTM densities (or more pathogenic species [25]), where certain regional environmental factors create a hospitable environment for NTM to persist. While previous literature has not identified molybdenum in soil or water as a risk factor for NTM, other surveyed metals have been identified as potential risk factors for NTM growth in the environment. In the coastal swamps of the southeastern US, high numbers of *M. avium*, *M. intracellulare*, and *M. scrofulaceum* were correlated with high zinc concentrations in water samples [26]. Although we did not observe an association between zinc concentrations in surface water and MAC infections, different NTM species may require specific environmental conditions for growth in different habitats, and thus discrepant findings are not unexpected. By analyzing water-quality data across diverse geographic regions, we hope to identify factors that are necessary in promoting NTM growth in water sources, as well as identifying whether these factors differ for MAC and *M. abscessus* species.

Figure 1 presents the predicted probability of *M. abscessus* infection by county. The more highly populated of these counties with the highest probability of *M. abscessus* infection, Denver, Mesa, Pueblo, and Weld, have public water supplies with centralized water distribution systems that come almost entirely from surface water sourced primarily from these rivers [27]. Among the rural counties with high probabilities of infection located along the South Platte River, the public water supply for Logan county is primarily from surface water, while Sedgwick county relies heavily on groundwater [27]. Many of the counties located along the major Colorado rivers also use water from these rivers for crop irrigation [27]. These county-level probabilities of infection suggest that potential sources of NTM exposure may come through municipal water systems that take water from these rivers as well as possibly from crop irrigation. The results shown in this map reflect the same high-risk regions that we have reported previously [11].

This study reports an important finding for the CF population. MAC and *M. abscessus* are the two most clinically relevant NTM species, which comprise 95% of NTM infections among pwCF [2, 3, 9, 28, 29]. Adjemian et al. observed significant increases for *M. abscessus* between 2010 and 2014 in the Mountain states among pwCF [2]. Rendering a framework of the necessary environmental factors that predict NTM exposure and infection is crucial for the development of prevention strategies.

### Strengths and limitations

In our previous studies [10, 11], we did not have sufficient data to identify and exclude individuals who had moved to Colorado after their initial infection diagnosis. The data used in this study ensured that a subject’s first positive culture occurred in Colorado, which prevented selection bias from influencing our results.

Only a subset of the water-quality constituent dataset for the state of Colorado was used in our analysis due to constraints in our study design. Counties were dropped from the analysis if no pwCF resided there. As a result, our findings were based on approximately half of Colorado’s counties. While our patient population included nearly all pwCF in Colorado, our results may therefore be generalizable to all pwCF in Colorado but only to the counties included in the analysis. In addition, some limitations are inherent to our water-quality constituent dataset [11]. Water sampling locations were not from random or systematically representative locations and the number of sites sampled across counties were variable. In addition, data were imputed to some counties with missing information. Therefore, we do not know the degree of bias in the resulting median concentration values for each county. If exposure misclassification with respect to water-quality constituents were present, we would assume it to be nondifferential with respect to cases and controls. This type of misclassification would bias the odds ratio toward the null. Finally, since source water samples were used in these analyses rather than tap water, our findings may not be representative of the water that people are exposed to in their homes after filtration and treatment.

## Conclusions

This study has identified molybdenum in surface water as the most predictive environmental factor of NTM infection among pwCF in Colorado, specifically for *M. abscessus* infection. We are too early in this discovery process to make specific recommendations, although if future studies confirm that molybdenum is in fact a necessary or sufficient factor for growth of *M. abscessus* species in water sources, these findings could inform patients at risk for NTM of their relative risks in residing within specific regions. Analyzing water-quality data across diverse geographic regions may render a framework of factors that are necessary for NTM growth, specifically factors that may differ for MAC and *M. abscessus* species. Investigating whether molybdenum metabolism in the (human) host affects NTM susceptibility will also have important implications for at-risk populations.

## Supplementary information

Supplementary information
