# Supplementary material for: Nontuberculous mycobacterial infection and environmental molybdenum in persons with cystic fibrosis: a case–control study in Colorado
Source: J Expo Sci Environ Epidemiol. 2021 Jul 3;32(2):289–94. doi: 10.1038/s41370-021-00360-2 (PMC8920885; doi:10.1038/s41370-021-00360-2)
Supplement: Supplementary file 1 — Supplementary information [file 41370_2021_360_MOESM1_ESM.docx]

Supplementary Table 1. Frequencies of NTM group species from patient isolates

| **Species groups diagnosed from patient isolates.** | **Culture-positive CF patients**  **n=193** |
| --- | --- |
| *M. abscessus, M. bolletii, M. chelonae* | 2 |
| *M. abscessus, M. chelonae* | 32 |
| *M. abscessus, M. chelonae, M. chimaera* | 1 |
| *M. abscessus, M. chimaera, M. chelonae, M. xenopi* | 1 |
| *M. abscessus, M. massiliense, M. chelonae* | 2 |
| *M. avium_complex* | 65 |
| *M. abscessus, M. avium_complex, M. chelonae* | 30 |
| *M. abscessus, M. avium_complex, M. chelonae, M. chimaera, M. massiliense* | 1 |
| *M. abscessus, M. avium_complex, M. chelonae, M. fortuitum* | 1 |
| *M. abscessus, M. avium_complex, M. chelonae, M. massiliense* | 4 |
| *M. avium_complex, M. chimaera* | 4 |
| *M. avium_complex, M. gordonae* | 5 |
| *M. avium_complex, M. gordonae, M. intracellulare* | 1 |
| *M. avium_complex, M. intracellulare* | 12 |
| *M. abscessus, M. avium_complex, M. chelonae, M. intracellulare* | 3 |
| *M. abscessus, M. avium_complex, M. chelonae, M. intracellulare, M. yongonense* | 1 |
| *M. avium_complex, M. intracellulare, M. chimaera* | 1 |
| *M. avium_complex, M. intracellulare, M. yongonense* | 2 |
| *M. avium_complex, M. lentiflavum* | 2 |
| *M. avium_complex, M. simiae* | 1 |
| *M. avium_complex, M. thermoresistible* | 1 |
| *M. avium_complex, M. chimaera, M. yongonense* | 3 |
| *M. abscessus, M. avium_complex, M. chelonae, M. chimaera, M. gordonae, M. lentiflavum* | 1 |
| *M. chimaera* | 2 |
| *M. abscessus, M. chelonae, M. gordonae* | 1 |
| *M. intracellulare* | 3 |
| *M. intracellulare, M. yongonense* | 1 |
| *M. kansasii* | 4 |
| *M. lentiflavum* | 2 |
| *M. abscessus, M. chelonae, M. lentiflavum* | 1 |
| *M. mucogenicum* | 1 |
| *M. abscessus, M. avium_complex, M. chelonae, M. fortuitum, M. massiliense, M. simiae, M. szulgai* | 1 |
| *M. chimaera, M. yongonense* | 1 |

Supplementary Table 2. Median and standard deviation (SD) values of water-quality constituents^*^ obtained from the Water Quality Portal (WQP) used in PCA.

| **Exposure Characteristics** | **Median ± SD** (µg/L) |
| --- | --- |
| Aluminum | 18 ± 4371.6 |
| Arsenic | <0.5 ± 49.9 |
| Cadmium | 0.1 ± 50.6 |
| Calcium | 32110 ± 70745.7 |
| Chloride | 2230 ± 219285.6 |
| Copper | 1.6 ± 440.8 |
| Iron | 38 ± 26245.6 |
| Lead | <0.5 ± 326.4 |
| Magnesium | 6691 ± 40822.9 |
| Manganese | 22.6 ± 7406.7 |
| Molybdenum | 4.3 ± 18.8 |
| Nickel | 1.2 ± 37.2 |
| Potassium | 1347 ± 6884.6 |
| Selenium | 0.06 ± 48.0 |
| Sodium | 6100 ± 123203.3 |
| Sulfate | 19000 ± 598707.4 |
| Zinc | 17 ± 5951.9 |

^*^The filtered portion (means the water was passed through a 0.45 micrometer filter) of the water-sample fractions were used.

Supplementary Table 3. Sensitivity analyses. Single-exposure Bayesian binomial regression model examining significant metals from Model 1 associated with odds of NTM infection among pwCF, excluding drive time. Bolded estimates have 90% CIs that fail to include 1. CI = Credible Interval.

| **All NTM species**  **Variable Odds Ratio**  **(95% CI)** | | **MAC species**  **Variable Odds Ratio**  **(95% CI)** | | **MABSC species**  **Variable Odds Ratio**  **(95% CI)** | |
| --- | --- | --- | --- | --- | --- |
| Age:  (1 Year) | 1.01  (1.00, 1.02) | Age:  (1 Year) | 1.01  (1.00, 1.03) | Age:  (1 Year) | 1.00  (0.98, 1.02) |
| Gender:  Male | 0.77  (0.54, 1.07) | Gender:  Male | 0.70  (0.48, 1.00) | Gender:  Male | 1.26  (0.81, 1.97) |
| Race:  Non-White^*^ | 0.78  (0.30, 1.97) | Race:  Non-White^*^ | 0.69  (0.22, 1.92) | Race:  Non-White^*^ | 1.26  (0.09, 1.51) |
| Molybdenum  (1-log unit) | 1.22  (0.99, 1.52) | Molybdenum  (1-log unit) | 1.19  (0.95, 1.48) | Molybdenum  (1-log unit) | **1.60**  **(1.22, 2.12)** |

^*^Reference group is White Alone

Supplementary Table 4. Bayesian binomial regression model examining the 11-contributing water-quality constituents from principal components 1 and 2 and other covariates associated with odds of NTM infection among pwCF. Bolded estimates have 90% CIs that fail to include 1. CI = Credible Interval.

| **All NTM species**  **Variable Odds Ratio**  **(95% CI)** | | **MAC species**  **Variable Odds Ratio**  **(95% CI)** | | **MABSC species**  **Variable Odds Ratio**  **(95% CI)** | |
| --- | --- | --- | --- | --- | --- |
| Age:  (1 Year) | 1.01  (1.00, 1.03) | Age:  (1 Year) | 1.01  (1.00, 1.03) | Age:  (1 Year) | 1.00  (0.98, 1.02) |
| Gender:  Male | 0.75  (0.53, 1.06) | Gender:  Male | 0.69  (0.47, 1.01) | Gender:  Male | 1.27  (0.79, 2.05) |
| Race:  Non-White^a^ | 0.82  (0.30, 2.12) | Race:  Non-White^a^ | 0.75  (0.23, 2.16) | Race:  Non-White^a^ | 0.38  (0.08, 1.46) |
| Drive-time  (>1.0 hours to NJH) | 1.37  (0.69, 2.64) | Drive-time  (>1.0 hours to NJH) | 1.57  (0.77, 3.06) | Drive-time  (>1.0 hours to NJH) | 1.26  (0.48, 3.10) |
| Calcium  (1-log unit) | **0.15**  **(0.02, 0.99)** | Calcium  (1-log unit) | 0.22  (0.02, 1.57) | Calcium  (1-log unit) | **0.02**  **(0.001, 0.22)** |
| Cadmium  (1-log unit) | 0.88  (0.52, 1.46) | Cadmium  (1-log unit) | 0.91  (0.53, 1.54) | Cadmium  (1-log unit) | 0.93  (0.42, 2.12) |
| Chloride  (1-log unit) | **0.42**  **(0.20, 0.81)** | Chloride  (1-log unit) | **0.43**  **(0.20, 0.90)** | Chloride  (1-log unit) | 0.41  (0.16, 1.02) |
| Magnesium  (1-log unit) | 1.91  (0.21, 19.5) | Magnesium  (1-log unit) | 1.07  (0.10, 12.4) | Magnesium  (1-log unit) | 11.48  (0.65, 247.2) |
| Manganese  (1-log unit) | 0.68  (0.35, 1.27) | Manganese  (1-log unit) | 0.64  (0.31, 1.23) | Manganese  (1-log unit) | 0.97  (0.41, 2.36) |
| Molybdenum  (1-log unit) | **2.89**  **(1.32, 6.89)** | Molybdenum  (1-log unit) | **2.54**  **(1.08, 6.49)** | Molybdenum  (1-log unit) | **7.11**  **(2.16, 25.5)** |
| Potassium  (1-log unit) | **5.56**  **(1.20, 30.9)** | Potassium  (1-log unit) | **5.75**  **(1.23, 33.1)** | Potassium  (1-log unit) | 7.69  (0.92, 76.7) |
| Selenium  (1-log unit) | 0.60  (0.33, 1.07) | Selenium  (1-log unit) | **0.55**  **(0.30, 0.98)** | Selenium  (1-log unit) | 0.96  (0.40, 2.32) |
| Sodium  (1-log unit) | 0.24  (0.03, 1.51) | Sodium  (1-log unit) | 0.43  (0.05, 3.16) | Sodium  (1-log unit) | **0.04**  **(0.002, 0.60)** |
| Sulfate  (1-log unit) | **8.23**  **(2.27, 37.3)** | Sulfate  (1-log unit) | **6.17**  **(1.54, 29.4)** | Sulfate  (1-log unit) | **20.0**  **(2.89 170.7)** |
| Zinc  (1-log unit) | 1.59  (0.79, 3.29) | Zinc  (1-log unit) | 1.36  (0.68, 2.83) | Zinc  (1-log unit) | 1.56  (0.54, 5.26) |

Supplementary Table 5. Correlation matrix (Pearson’s Correlation Coefficient, ρ) for the water-quality constituents contributing to Principal Components 1 & 2.

|  | Cd | Ca | Cl | Mg | Mn | Mo | K | Se | Na | SO_4_^2-^ | Zn |
| --- | --- | --- | --- | --- | --- | --- | --- | --- | --- | --- | --- |
| Cadmium  (Cd) | 1.00 |  |  |  |  |  |  |  |  |  |  |
| Calcium  (Ca) | 0.22 | 1.00 |  |  |  |  |  |  |  |  |  |
| Chloride  (Cl) | 0.13 | 0.78 | 1.00 |  |  |  |  |  |  |  |  |
| Magnesium  (Mg) | 0.31 | 0.96 | 0.77 | 1.00 |  |  |  |  |  |  |  |
| Manganese  (Mn) | 0.50 | 0.59 | 0.46 | 0.53 | 1.00 |  |  |  |  |  |  |
| Molybdenum (Mo) | -0.06 | 0.70 | 0.69 | 0.69 | 0.38 | 1.00 |  |  |  |  |  |
| Potassium  (K) | 0.28 | 0.88 | 0.80 | 0.88 | 0.65 | 0.76 | 1.00 |  |  |  |  |
| Selenium  (Se) | -0.17 | 0.72 | 0.68 | 0.70 | 0.32 | 0.78 | 0.75 | 1.00 |  |  |  |
| Sodium  (Na) | 0.25 | 0.88 | 0.82 | 0.91 | 0.59 | 0.82 | 0.95 | 0.76 | 1.00 |  |  |
| Sulfate  (SO_4_^2-^) | 0.22 | 0.90 | 0.88 | 0.90 | 0.52 | 0.64 | 0.82 | 0.67 | 0.87 | 1.00 |  |
| Zinc  (Zn) | 0.58 | 0.27 | 0.26 | 0.26 | 0.60 | 0.14 | 0.25 | 0.07 | 0.26 | 0.28 | 1.00 |

Supplementary Table 6. Summary of posterior probability that the Molybdenum covariate is associated with increased odds of NTM infection among pwCF.

| **All NTM species** | **MAC species** | **MABSC species** |
| --- | --- | --- |
| 96.96% | 94.15% | 99.96% |

Supplementary Figure 1. Contribution of water-quality constituents to principal components 1 and 2.

Supplementary Figure 2. Directed Acyclic Graph (DAG) depicting the relationship between confounders, exposure and dependent variables.

**Supplementary Methods**

**2.2 Statistical Analysis**

Analysis of data was performed using the R packages: **rgdal** (1), **sp** (2), **rstanarm** (3), **dplyr** (4), **standardize** (5), **missMDA** (6),**gmapdistance** (7), **FactoMineR** (8), and **factoextra** (9). All water-sample sites were aggregated by county using the **sp** package. We calculated the median value of each water-quality constituent for each county using the **dplyr** package. The R source code that we created to calculate the county medians is available in the Supplementary Materials. Using the scale function from the **standardize** package, we standardized all the water-quality constituents’ log concentrations to have a mean of 0 and standard deviation of 1. Using the scale function from the **standardize** package, we standardized all the water-quality constituents’ log concentrations to have a mean of 0 and standard deviation of 1. For counties with missing data, we imputed the median value of all water-quality constituents using the imputePCA function in the **missMDA** package. Drive time between county centroids and NJH were calculated using the R **gmapsdistance** package.

*2.2.1 Variable Reduction using Principal Component Analysis (PCA)*

PCA was performed using the PCA function in the **FactoMineR** package on 17 water-quality constituents summarized at the county-level (after these values were natural log transformed, scaled, and imputed). We used the fviz_contrib function in the **factoextra** package to identify the most important variables in explaining variability of principal components 1 and 2.

*2.2.3 Parameters used in Bayesian Binomial Regression Models*

We used Bayesian generalized linear models (GLM) to model the response as Binomial, which links the logit of the probability of NTM occurrence to a weighted linear combination of the predictors via the **rstanarm** package (3).

For the prior distribution of the intercept, we used a Student’s t distribution with a 1 degree of freedom, a location parameter of 0 and a scale parameter of 2.5 For the prior distributions of the remaining regression coefficients, we used independent and identically-distributed normal distributions with a mean of 0 and a standard deviation of 5. Our models assumed overdispersed, binomial-distributed discrete responses and used the logit link function; the posterior distributions were approximated using 10,000 Markov chain Monte Carlo (MCMC) iterations, which includes a default warmup period of 5,000 iterations.

*2.2.4 Bayesian Binomial Regression Models with Individual Metals from Principal Components 1 & 2.*

The posterior probabilities shown in Table 4 (Model 3) were calculated using the **rstanarm** and **rstan** packages (3, 10). We used the posterior_linpred function from the **rstanarm** R package to predict the probability that an unobserved CF patient living in a county will have an NTM infection and displayed the results as a probability map across Colorado counties (Figure 1).

References:

1. Bivand R, Keitt T, Rowlingson B. rgdal: Bindings for the 'Geospatial' Data Abstraction Library. R package version 1.5-12. 2020. https://CRAN.R-project.org/package=rgdal.

2. Roger S, Bivand EP, Virgilio Gomez-Rubio. Applied spatial data analysis with R. Springer, NY2013.

3. Goodrich B Gabry J, Ali I, Brilleman S. rstanarm: Bayesian applied regression modeling via Stan. R package version 2.19.3. 2020.

4. Wickham H, Francois R, Henry L, Muller K. dplyr: A Grammar of Data Manipulation. R package version 083 2019.

5. Eager CD. standardize: Tools for Standardizing Variables fo Regression in R. R package version 0.2.1. 2017.

6. Josse J, Husson, F. missMDA: A Package for Handling Missing Values in Multivariate Data Analysis. Journal of Statistical Software. 2016;70(1):1-31.

7. Azuero Melo R, Rodriguez D, Zarruk D. gmapsdistance: Distance and Travel Time Between Two Points from Google Maps. R package version 3.4. 2018.

8. Le S, Josse J, Husson F. FactoMineR: An R Package for Multivariate Analysis. Journal of Statistical Software. 2008;25(1):1-18.

9. Kassambara A, Mundt F. factoextra: Extract and Visualize the Results of Multivariate Data Analyses. R package version 1.0.7. 2020.

10. Team SD. RStan: the R interface to Stan. R package version 2.19.3. 2020.
